# Pre-hospital extra-corporeal cardiopulmonary resuscitation

**DOI:** 10.1186/s13049-018-0489-y

**Published:** 2018-03-27

**Authors:** Ben Singer, Joshua C. Reynolds, David J. Lockey, Ben O’Brien

**Affiliations:** 1 St Bartholomew’s Hospital and Barts Heart Centre, Barts Health NHS Trust, London, UK; 2 Department of Emergency Medicine, Michigan State University College of Human Medicine, Grand Rapids, MI USA; 3 The Blizard Institute, Queen Mary University, London, UK; 4 William Harvey Research Institute, Queen Mary University, London, UK; 5 Outcomes Research Consortium, Cleveland, OH USA

**Keywords:** Pre-hospital, Extracorporeal cardiopulmonary resuscitation, Extracorporeal membrane oxygenation, Extracorporeal life support

## Abstract

Survival from out-of-hospital cardiac arrest (OHCA) has remained low despite advances in resuscitation science. Hospital-based extra-corporeal cardiopulmonary resuscitation (ECPR) is a novel use of an established technology that provides greater blood flow and oxygen delivery during cardiac arrest than closed chest compressions. Hospital-based ECPR is currently offered to selected OHCA patients in specialized centres. The interval between collapse and restoration of circulation is inversely associated with good clinical outcomes after ECPR. Pre-hospital delivery of ECPR concurrent with conventional resuscitation is one approach to shortening this interval and improving outcomes after OHCA. This article examines the background and rationale for pre-hospital ECPR; summarises the findings of a literature search for published evidence; and considers candidate selection, logistics, and complications for this complex intervention.

## Backround and rationale

Survival to discharge from out-of-hospital cardiac arrest (OHCA) remains poor. In London overall reported survival is 9% for patients with out-of-hospital cardiac arrest and attempted resuscitation (31.5% for witnessed cardiac arrest with initial shockable rhythm) [1]. This mirrors a reported global OHCA survival rate of 2–11% with corresponding regional variation [2]. During conventional resuscitation, external chest compressions generate both coronary perfusion pressure and cardiac output [3, 4]. Coronary perfusion pressure dictates myocardial reperfusion, which in turn is critical to achieve return of spontaneous circulation (ROSC) [4]. Cardiac output dictates organ and cerebral perfusion, which is critical to prevent irreversible ischemic injury. The organ and cerebral perfusion delivered is influenced by a variety of factors, including the quality of chest compressions, body habitus, underlying comorbidities, and the aetiology of cardiac arrest. Optimal conventional cardiopulmonary resuscitation (CCPR) typically generates only a fraction of normal cardiac output (cardiac index ~ 0.6 L.min-1.m-2), commonly referred to as the ‘low-flow’ state [5]. A lengthy low-flow state during prolonged CCPR increases the risk of multi-organ failure and hypoxic brain injury after ROSC.

Extra-corporeal cardiopulmonary resuscitation (ECPR) is the implementation of veno-arterial extracorporeal membrane oxygenation (VA-ECMO) during ongoing resuscitation attempts in cardiac arrest. In VA-ECMO, a venous drainage cannula is placed to drain blood which is then pumped through a membrane oxygenator before being returned under pressure into a central artery through a return cannula. There are various configurations of where cannulae can be placed, however the most common selection in cardiac arrest is a femoral vein drainage cannula and a femoral artery return cannula. Compared to CCPR, ECPR improves blood flow (cardiac index ~ 2.0 L.min-1.m-2) and oxygen delivery during cardiac arrest with the aim of preventing irreversible end-organ damage and hypoxic brain injury. It can also facilitate therapies such as coronary angiography or fibrinolysis to treat the primary cause of OHCA [6]. Although there are currently no published randomised trials of ECPR, observational evidence supports its use in carefully selected candidates [7].

A primary determinant of successful clinical outcomes after ECPR is the interval between collapse and onset of ECPR. This interval is further divided into ‘no-flow time’, the interval between collapse and onset of external chest compressions, and ‘low-flow time’, the interval between external chest compressions and onset of ECPR. Observational studies of ECPR for in-hospital cardiac arrest (IHCA) and OHCA highlight the importance of brief low-flow times. Survival to hospital discharge after ECPR for IHCA ranges from 20 to 35% [8, 9], whereas survival to hospital discharge after ECPR for OHCA is approximately 15% [10]. A key difference between these two populations is the longer low-flow intervals of CCPR in OHCA (as high as 80–155 min) [11–15]. A recent systematic review confirms that longer intervals of conventional resuscitation preceding ECPR (low-flow time) are associated with poor clinical outcomes (geometric mean ratio 0.90; 95% CI 0.81–0.99) [10].

During OHCA resuscitation, pre-hospital emergency care providers concentrate on optimizing CCPR and advanced life support, achieving ROSC, and identifying reversible causes of cardiac arrest. In the United Kingdom, CCPR typically lasts for a minimum of 20 min before resuscitation is terminated or the patient is transported to hospital with ongoing CPR [16]. Indeed, many guidelines advocate at least 20 min of resuscitation [17, 18]. Pre-hospital ECPR seeks to minimize low-flow time by delivering ECPR to the patient concurrently with initial CCPR. While posing unique logistical challenges, this strategy may shorten the delay to commencement of ECPR.

## Hospital-based vs. pre-hospital ECPR for OHCA

In pre-hospital systems of care there is historical emphasis on resuscitation of OHCA patients on scene compared to the alternative strategy of early transport to hospital with ongoing resuscitation. This philosophy of remaining on scene is driven by the recognition that the best outcome for a patient in cardiac arrest is early ROSC, and the primary drivers of favourable outcome are high quality CPR and the treatment of reversible pathology. ROSC is most likely within the first few minutes of resuscitation, and if ROSC has not been achieved by 15 min of resuscitation there is only a 10–15% chance of subsequent good neurologic outcome [19, 20]. Since CPR quality degrades during patient extraction and transport to hospital, [21] many pre-hospital systems of care mandate a minimum resuscitation interval on scene before consideration of transport with CPR in progress. The only treatment offered at most hospitals is additional CCPR, and only 3–4% of patients survive when transported to hospital without ROSC [22, 23]. Some hospitals will consider fibrinolysis or coronary angiography with percutaneous coronary intervention in highly selected OHCA cases with ongoing CCPR [24]. Otherwise, ECPR offers the only novel additional therapy for patients transported to hospital after CCPR has failed. Hospital-based ECPR does offer a more controlled environment with immediate access to invasive monitoring, additional diagnostics, and additional therapeutic interventions to treat the underlying aetiology of cardiac arrest. However, hospital-based ECPR necessitates patient extraction, packaging, and transport, which all contribute to additional low-flow time and risk of subsequent multi-organ failure and hypoxic brain injury.

The ideal therapeutic window for ECPR is within 60 min after collapse [25]. Patients most likely to benefit from ECPR (e.g. younger age, witnessed, bystander CPR, shockable rhythm) also have the greatest probability of ROSC with CCPR during the first 10–15 min after collapse [20]. Pre-hospital systems of care with hospital-based ECPR need to factor in the time required for initial resuscitation efforts with CCPR on scene, time required for extraction and transport to hospital, the logistics of maintaining quality chest compressions en route to the hospital, and the procedural time needed to cannulate and initiate VA-ECMO. Some systems employ mechanical chest compression devices with early transport of potential ECPR patients to mitigate the degradation in CCPR quality and still allow sufficient time for VA-ECMO initiation at hospital [26]. Other systems deliver pre-hospital ECPR to the patient, eliminating the problems of CCPR during transport and attempting to decrease low-flow time compared to hospital-based ECPR [27].

## Published evidence

We conducted a literature search of Medline to include English language papers that described the pre-hospital implementation of extracorporeal cardiopulmonary resuscitation. Search terms used were *extracorporeal cardiopulmonary resuscitation*, *prehospital* and *extracorporeal life support* and *extracorporeal membrane oxygenation*. In total, we reviewed 1108 titles to identify 65 potential abstracts that ultimately yielded six publications. We identified one additional case series abstract through a semi-structured internet search using the same search terms. Table 1 summarizes the published literature to date on prehospital ECPR.

**Table 1:** Published literature on pre-hospital ECPR

Reference	Type of study	No. pre-hospital ECPR patients	Mean low flow interval
Arlt, et al. 2011 [47]	Case study	1	> 90 min
Lebreton, et al. 2011 [48]	Case study	1	60 min
Lamhaut, et al. 2012 [49]	Case study	1	60 min
Lamhaut, et al. 2013 [46]	Case series	7*	79 min
Hilker, et al. 2013 [50]	Case series	6	61 min
Lamhaut, et al. 2017 [51]	Case study	1	90 min
Lamhaut, et al. 2017 [27]	Before-and-after cohort study	46 (period 1) 27 (period 2)**	93 min (period 1)*** 71 min (period 2)***

*includes case reported in Lamhaut, et al. 2012. ** includes case reported in Lamhaut, et al. 2017. *** mean duration for all ECPR patients (47% prehospital ECPR vs. 53% hospital-based ECR). ECPR: extracorporeal cardiopulmonary resuscitation; min: minutes

Most published literature for pre-hospital ECPR comprises case studies and small case series. In total we found 88 reported cases of the pre-hospital implementation of ECPR with an overall survival rate of 15% (13/88). The sole cohort study by Lamhaut et al. in 2017 compared 2 periods of ECPR strategy in a before-and-after fashion. The initial strategy (period 1) involved a mandatory 30 min interval of CCPR before either transport to hospital (if within 20 min range) or initiation of pre-hospital ECPR. These logistical constraints resulted in a typical low-flow interval up to 90 min in duration, and this approach to pre-hospital ECPR yielded 8% survival with good neurological outcome. For context, the longest low-flow duration for any neurologically intact survivor in a recent multi-centre North American cohort of > 11,000 OHCA patients was 47 min [20]. The revised strategy (period 2) entailed a variety of modifications, notably a decision to initiate ECPR within 20 min of CCPR, and a greater emphasis on pre-hospital delivery of ECPR (unless the hospital was within 10 min range). Additionally, the selection criteria were more stringent, the ECPR team was dispatched as primary response to all OHCA cases < 70 years old, and epinephrine dosing was limited to a maximum of 5 mg. This revised strategy improved survival with good neurologic outcome to 29% (21% absolute increase), and the mean low-flow interval was reduced by 20 min. In propensity-matched analysis, pre-hospital ECPR had shorter low-flow intervals and higher rates of ROSC compared to hospital-based ECPR. There was no difference in survival, but the authors note the risk of a survival time bias in the hospital-based ECPR patients who had additional low-flow period during extraction, packaging, and transport to hospital [27].

### Forthcoming prehospital ECPR trials

The first randomised prospective trial comparing two different strategies of delivering ECPR to OHCA patients (prehospital ECPR vs. hospital-based ECPR) is currently recruiting in France (ACPAR2; NCT02527031) with an estimated completion date of March 2019. Patients randomized to prehospital ECPR will receive ECPR between 20 and 30 min after collapse at the site of collapse. Those randomized to hospital-based ECPR will receive on-site CCPR and transfer to hospital for subsequent ECPR. The investigators hypothesize that a shorter low-flow period in the prehospital ECPR arm will translate into superior survival with good neurologic outcome. Selection criteria include a no-flow time < 5 mins, age 18–65 years, refractory arrest defined as 20 min of CCPR, and presence of shockable rhythm or signs of life during resuscitation [28]. A search of clinicaltrials.gov demonstrates no other current pre-hospital ECPR trials. There are three other hospital-based ECPR trials (NCT01511666, NCT02832752, NCT03065647) that may yield data that is extrapolated to pre-hospital ECPR.

## Patient selection

Successful outcomes after ECPR largely depend on appropriate candidate selection. Most OHCA patients will not be candidates for pre-hospital ECPR. Multiple observational studies have identified consistent prognostic factors in OHCA patients that are most likely to benefit from hospital-based ECPR. The precise sensitivity and specificity of each of these criteria are still undefined, as is the product of relaxing specific criteria. These prognostic factors can be extrapolated to select suitable candidates for pre-hospital ECPR (Table 2). Relaxation of these inclusion criteria may yield additional patients that benefit from pre-hospital ECPR, but will almost certainly reduce the overall neurologically intact survival rate. Absent from this list is some evidence-based consideration of baseline functional status, including overall health, comorbidities, quality of life, cognition, independence, etc. These aspects are important, but there is little data to guide clinicians towards a reliable and valid means to assess these intangible factors in the acute setting when considering ECPR.

**Table 2:** Suggested Criteria for Pre-Hospital ECPR Selection

Inclusion Criteria for Consideration of Pre-hospital ECPR:	
1. Age 18–65 years	
2. Witnessed arrest with bystander CPR	
3. VF/VT Rhythm or signs of life during resuscitation*	
4. No-flow time < 5 min	
5. Ability to initiate ECPR within 60 min of collapse	

*signs of life include attempted respiratory effort, gasps, movement, or pupil reactivity. *ECPR* extracorporeal cardiopulmonary resuscitation, *CPR* cardiopulmonary resuscitation, *VF* ventricular fibrillation, *VT* ventricular tachycardia

### Age

Age is an established prognostic factor in OHCA after CCPR. There is an inverse relationship between age and likelihood of survival or good neurologic outcome, and a progressive decline in likelihood of good outcome after 64 years [29]. Several ECMO centres have used an upper age limit of 75 years as a selection criterion for hospital-based ECPR [30–32]. A recent Parisian pre-hospital OHCA ECPR cohort study used an upper age limit of 70 years, although the median age of patients was 51 years and only 23% of patients were more than 60 years old [27]. An Australian mixed IHCA and OHCA cohort used an upper age limit of 65 years and the median age of patients was 52 years [26]. An association between age and favourable outcome has not yet been established in ECPR observational studies for OHCA [10]. It is often difficult to accurately assess age in OHCA. Until better evidence is available, 65 years appears to be a reasonable upper limit for pre-hospital ECPR. (We do note the distinction between ‘chronologic’ age and ‘physiologic’ age as a function of comorbidities and overall health, and acknowledge that prehospital ECPR could benefit some patients older than 65 years). Hospital-based ECPR is employed in paediatric patients, most commonly those with IHCA and/or known heart disease [33, 34]. There is no evidence on the inclusion of paediatric patients in pre-hospital ECPR programs. While the anatomy and physiology of some teenagers may be similar to those of young adults, at some point inclusion of younger age groups requires additional paediatric-specific training in VA-ECMO.

### Witnessed arrest and bystander CPR

Witnessed collapse and bystander CPR are both positive prognostic factors in OHCA after CCPR [35], especially in cases of prolonged resuscitation [19]. These prognostic factors have incompletely translated to ECPR, because firm conclusions are limited by the small number of studies and variable reporting methods [10]. Both factors highlight the issue of ‘no-flow’ time, which is critical because neuronal cell death begins within minutes of loss of cerebral oxygen delivery [36]. Most ECPR centres use unwitnessed events or ‘no-flow’ interval > 5 min as exclusion criteria [27, 32]. The case details of witnessed collapse and bystander CPR can often be established on scene, so witnessed collapse with initiation of bystander CPR within 5 min are reasonable selection criteria for pre-hospital ECPR.

### Cardiac rhythm

Shockable initial cardiac rhythm (ventricular fibrillation or pulseless ventricular tachycardia) is a major prognostic factor for OHCA and has been used as an inclusion criterion for ECPR [12]. A recent systematic review found that shockable initial cardiac rhythm is associated with favourable clinical outcomes after ECPR for OHCA (summary odds ratio 2.20; 95% CI 1.30–3.72) [10]. Asystole has significant negative prognostic value, and is often an exclusion criterion for ECPR. Pulseless electrical activity (PEA) represents a clinical challenge to the clinician. It is often grouped together with asystole as a non-shockable rhythm, and is commonly regarded as a negative prognostic factor. However PEA cardiac arrests of certain aetiologies are reversible and carry a good prognosis with ECPR (e.g. pulmonary embolism, environmental hypothermia) [37, 38]. Additionally, PEA can actually reflect different physiologic states: complete electro-mechanical dissociation with cardiac standstill and residual electrical activity, or impaired circulation with preserved cardiac motion but no palpable pulses. The former is unlikely to respond to ECPR, but the latter is very treatable with ECPR given a reversible aetiology. A pragmatic approach may be to include any subject with organized electrical activity and exclude asystole. Alternatively, patients with signs of life (e.g. respiratory efforts or agonal breaths, patient movement, or pupillary light reactivity) could be included irrespective of rhythm. The latter combination of selection criteria was utilized in the recent Parisian pre-hospital ECPR cohort study [27]. Notably, in that cohort there were no ECPR survivors that did not demonstrate some signs of life during CCPR prior to ECPR initiation.

### Low flow duration

The prognostic value of low-flow duration is well established for ECPR after both IHCA and OHCA. Among IHCA cases, overall survival after ECPR was 30–40% with low-flow times < 60 min, and only 15–20% with low-flow times > 60 min [25, 30]. A recent systematic review found that low-flow duration was inversely associated with favourable outcome after ECPR (summary geometric mean ratio 0.90; 95% CI 0.81–0.99) [10]. Low-flow time may be the most important factor that differentiates the higher survival after ECPR observed for IHCA compared to OHCA [39]. This is consistent with the strong physiological argument that a longer low-flow time risks irreversible multi-organ failure and hypoxic brain injury, negating the potential benefits of ECPR. A collapse to ECPR interval no longer than 60 min is a common selection criterion at many ECMO centres [40, 41]. Since the fundamental justification for pre- hospital ECPR is reducing the low-flow interval, it should be as brief as possible and not exceed 60 min.

## Timing of ECPR initiation

Given the adverse effects of ‘low-flow’ time, it would seem reasonable to initiate ECPR as soon as possible after collapse in eligible candidates. However, initiating ECPR too early in the resuscitation exposes patients to invasive procedures with significant complications and unproven outcome benefits when a significant proportion will achieve ROSC within the initial minutes of CCPR. Furthermore, distracting from the emphasis on continuous, high-quality chest compressions is potentially harmful [42]. Yet traditional resuscitation most often fails, and the likelihood of ROSC steadily decreases with elapsed durations of CCPR [43]. At what elapsed interval of CCPR do the potential benefits of ECPR outweigh the procedural risks and the likelihood of failed CCPR? Common practice is to require 20–30 min of CCPR before declaring OHCA ‘refractory’ and initiating ECPR [27, 44]. However, based on the natural history of large North American cohorts of OHCA cases, it is reasonable to shift from CCPR to ECPR after 10–20 min of CCPR [19, 20]. This window strikes the best balance between maximizing outcomes with CCPR (~ 90% of patients with eventual good neurologic outcome had achieved ROSC) and recognizing the time constraints of the therapeutic window for ECPR.

## Logistical considerations

The logistics for providing a pre-hospital ECPR service are complex and intimidating. For optimal results and the shortest low-flow times, the pre-hospital ECPR team should be a primary response to out-of-hospital cardiac arrests (i.e. dispatched at the time of the initial emergency service call, rather than as a secondary response once cardiac arrest has been confirmed by on-scene emergency services) [27]. This necessitates screening all collapse, unresponsive, and cardiac arrest calls made to emergency services, identifying those calls most likely to meet inclusion criteria, and dispatching an ECPR team concurrently with standard pre-hospital resources. Given the limited pool of ECPR-trained personnel, a single ECPR team may have to serve a large geographical area. For example, London Ambulance Service attended 10,116 out-of-hospital cardiac arrests in 2015–2016. Of these, 560 (5.5%) were witnessed events with bystander CPR and an initially shockable cardiac rhythm – cases most likely to meet ECPR selection criteria [45]. This provides a tremendous challenge for dispatchers trying to identify the approximate 1 in 20 arrests that meet only some of the criteria for consideration of ECPR. In order to capture suitable cases, some degree of over-triage is inevitable. Thus, the logistics of pre-hospital ECPR may be most suited to an urban environment where a fast response ground vehicle could transport the ECPR team and equipment with a target response time within 10 min. However, given the resources required, prehospital ECPR is likely not practical or even feasible in all metropolitan regions. The rural environment provides significant barriers to pre-hospital ECPR, where ground-based response frequently lasts 20–30 min or longer to get to scene, negating the reduction in low-flow interval sought with pre-hospital ECPR. Integration with helicopter emergency medical teams (HEMS) and transport by rotatory wing aircraft may achieve faster dispatch-to-scene intervals, but would be a substantial resource burden given the inevitable over-triage of the ECPR team to cases ultimately not suitable for ECPR. The selective targeting of large population high risk events such as marathons with an ECPR team is a potential trade off between resource allocation and likely patient benefit.

The financial costs of pre-hospital ECPR are high: this includes both fixed and variable costs of equipment and personnel, as well the expectation that there will be attendance at OHCA cases ultimately deemed not suitable for ECPR. The required personnel for pre-hospital ECPR varies at different institutions, but would commonly include at a minimum two consultant-level specialists and a clinical perfusionist. Future anticipated technological advancements and concurrent reductions in the complexity of priming a VA-ECMO circuit and preparing a console may allow change in both the required seniority of specialists and number of team members. However, the availability of pre-hospital physicians integrated into the emergency medical response will likely be a pre-requisite for such a complex intervention, limiting pre-hospital ECPR implementation to countries and healthcare systems where this occurs.

We have estimated costs of approximately 880,000 Euros a year to provide an equipped primary response ECPR team for 9 h a day, 7 days a week. Other cost considerations include the environmental and human factor issues involved in the pre-hospital environment. Hospital-based physicians may not be used to performing complex clinical tasks and procedural steps in a variety of pre-hospital environments, and may struggle to integrate into established pre-hospital emergency response teams. Such procedural tasks include establishing a sterile field and cannulating femoral vessels. Percutaneous and surgical cut-down techniques have been described, and the choice of technique may be made on clinical circumstance and team experience. After insertion, optimizing circulatory flow and ensuring adequate systemic vascular resistance is challenging in the absence of invasive hemodynamic monitoring. Pre-hospital ECPR teams may have to titrate circuit settings and medication infusions to venous oxygenation saturations until arrival at hospital. Pre-hospital and human factor training may be necessary, which adds additional costs and resources.

Discussion of cost raises the larger economic questions of whether pre-hospital ECPR is more or less cost effective than other endeavours to improve survival after OHCA, including public education campaigns to increase the prevalence of bystander CPR provision and use of an automated external defibrillator (‘AED’). Direct comparisons of cost effectiveness are not possible until the cost effectiveness of ECPR has been fully evaluated. As with any other complex and costly intervention, opportunity cost ought to be discussed.

## Complications

There are many anticipated complications when delivering a complex procedure in the pre-hospital environment (Table 3). All hospital-based complications of ECPR are present along with additional risks specific to the pre-hospital environment. The sole pre-hospital ECMO-related complication reported to date is a case of accidental cannula displacement [46].

**Table 3:** Common complications of prehospital ECPR

Complication	Specific Pre-hospital Concerns
Vascular injury and Bleeding	Availability of pre-hospital blood products, difficulty recognising complications such as retroperitoneal bleeding. No access to interventional radiology or operating theatres.
Failure to cannulate	Hospital-based percutaneous VA-ECMO cannulation has a reported failure rate between 7% and 10% [52, 53] and is anticipated to be higher in the pre-hospital environment. Surgical cut down may reduce the expected failure rate in the pre-hospital setting.
Limb Ischaemia	In-hospital limb ischaemia after insertion of VA-ECMO cannulae is reported in the range of 12–15% [31, 52] and would be similar in the pre-hospital environment. The usual practice of inserting a retrograde distal limb perfusion cannula would be deferred until arrival at hospital. One alternative could be using smaller calibre arterial cannulae accepting either lower flows or higher pressures.
Infection	Although the true infection rate related to ECMO cannulae insertion is unknown, ECMO is an independent risk factor of blood stream infection. [54] Pre-hospital ECMO insertion will not be as clean as an operating theatre and the infection risk may be increased.

*ECPR* extracorporeal cardiopulmonary resuscitation, *VA-ECMO* veno-arterial extracorporeal membranous oxygenation, *ECMO* extracorporeal membranous oxygenation

## Conclusion

Pre-hospital ECPR seeks to address the physiological rationale and observational data supporting the reduction of low-flow time as much as possible to improve survival and good functional outcome after OHCA. Forthcoming prospective trials of pre-hospital ECPR (NCT02527031) and hospital-based ECPR (NCT01511666, NCT02832752, NCT03065647) will aid in refining selection criteria and identifying which patients may benefit from pre-hospital ECPR compared to hospital-based ECPR or CCPR. The ideal dispatch and deployment strategy for pre-hospital ECPR teams has yet to be determined, and will likely depend on unique features of each pre-hospital system of care. The clinical benefits of rapid response and shorter low-flow time need to be balanced with the costs of over-triage and resource utilization. A deeper understanding of the health economics surrounding pre-hospital ECPR is also required to justify the large resource requirement. In summary, more evidence is required and regional systems of care deploying pre-hospital ECPR are encouraged to publish prospectively collected data on their protocols, process measures, and outcomes. Despite its challenges, pre-hospital ECPR offers the potential to dramatically improve clinical outcomes for a subset of OHCA patients.

## Abbreviations

CCPR: Conventional Cardiopulmonary Resuscitation
CPR: Cardio-pulmonary Resuscitation
ECLS: Extracorporeal Life Support
ECMO: Extracorporeal Membrane Oxygenation
ECPR: Extracorporeal Cardio-pulmonary Resuscitation
ELSO: Extracorporeal Life Support Organisation
IHCA: In Hospital Cardiac Arrest
OHCA: Out of Hospital Cardiac Arrest
PCI: Percutaneous Coronary Intervention
ROSC: Return of Spontaneous Circulation
VA-ECMO: Veno-arterial Extracorporeal Membrane Oxygenation
VF: Ventricular Fibrillation
VT: Ventricular Tachycardia
